# Women’s experiences and perspectives on a nurse navigation program for cervical cancer screening: a qualitative study

**DOI:** 10.3389/fpubh.2026.1883702

**Published:** 2026-07-01

**Authors:** Turkan Ozdas, Elif Donmez, Tulay Ortabag

**Affiliations:** 1 Radiotherapy Program, Vocational School of Health Services, Fenerbahçe University, Istanbul, Türkiye; 2 Department of Oncology Nursing, Hamidiye Faculty of Nursing, University of Health Sciences, İstanbul, Türkiye; 3 Department of Nursing, Faculty of Health Sciences, Istanbul Topkapi University, İstanbul, Türkiye

**Keywords:** cervical cancer screening, health belief model, HPV self-sampling, nurse navigation, thematic analyses, women’s health

## Abstract

**Background:**

Cervical cancer remains an important public health problem despite the availability of effective screening and prevention strategies. Psychosocial, individual, and health system-related barriers continue to limit women’s participation in cervical cancer screening programs. Recent approaches increasingly emphasize the role of nurse navigation, digital health technologies, media communication, and HPV self-sampling strategies in improving access to preventive healthcare services and promoting screening participation. Therefore, this study aimed to explore in depth the experiences and perspectives of women who participated in a nurse navigation program regarding cervical cancer screening.

**Methods:**

This thematic qualitative study was conducted in Istanbul, Türkiye, with women who had participated in a nurse navigation program and undergone cervical cancer screening. Participants were selected using purposive sampling. Data were collected through semi-structured in-depth interviews and analysed using thematic analysis. The reporting of the study was guided by the COREQ checklist.

**Results:**

Twelve women participated in the study. The findings demonstrated that the nurse navigation program improved women’s knowledge and awareness regarding HPV, cervical cancer, and screening. However, psychosocial barriers such as fear, shame, embarrassment, and avoidance of hospital environments continued to influence screening behavior. Participants generally expressed positive attitudes toward HPV self-sampling, media-based awareness strategies, and artificial intelligence–supported health information systems. Individual health perceptions, social support, and health system-related factors were also found to shape women’s participation in screening programs.

**Conclusion:**

The findings suggested that participants perceived the nurse navigation program as contributing to increased knowledge and awareness regarding HPV, cervical cancer, and screening. Nurse navigation programs may contribute to improving screening participation by providing education, guidance, and supportive communication. Multidimensional and community-based approaches addressing both individual and structural barriers may enhance participation in cervical cancer screening programs.

## 1 Introduction

Cervical cancer remains one of the most important public health problems affecting women worldwide and continues to cause significant morbidity and mortality despite advances in prevention and treatment (1). It is the fourth most common cancer among women globally, with an estimated 660,000 new cases and approximately 350,000 deaths reported in 2022 (1, 2). Persistent infection with high-risk types of human papillomavirus (HPV) has been identified as the primary cause of cervical cancer, accounting for nearly all cases worldwide (1). Although cervical cancer is largely preventable through vaccination, screening, and early treatment of precancerous lesions, the disease burden remains disproportionately high in many regions due to inequalities in access to preventive services and health education (2, 3).

Cervical cancer screening plays a critical role in reducing both the incidence and mortality of the disease by enabling early detection and treatment of precancerous lesions before they progress to invasive cancer (3). Screening methods such as the Papanicolaou (Pap) smear and human papillomavirus (HPV) testing have been shown to significantly decrease cervical cancer incidence and mortality when implemented within organized screening programs (4, 5). For this reason, many international health organizations recommend regular cervical cancer screening for women within specific age groups as a key strategy for cervical cancer prevention (1, 6, 7).

However, despite these recommendations, participation in cervical cancer screening programs remains insufficient in many countries worldwide (3, 8). Studies have shown that multiple factors contribute to low screening uptake, including lack of knowledge about cervical cancer and screening, fear of diagnosis, embarrassment, cultural beliefs, and limited access to health services (9, 10). Socioeconomic inequalities, low health literacy, and inadequate awareness further reduce women’s participation in preventive health services (11). In Türkiye, although a population-based cervical cancer screening program has been implemented through primary health care services and Cancer Early Diagnosis, Screening and Education Centers (KETEM), screening participation among eligible women remains below the desired level (12, 13).

Recent global evidence highlights the ongoing challenges in cervical cancer screening participation. A study including data from 202 countries reported that only 36% of women aged 30–49 years had undergone cervical cancer screening at least once in their lifetime, with this proportion decreasing to 9% in low- and middle-income countries (6). Similarly, national data from Türkiye indicate that 64.4% of women have never undergone a Pap smear test, while reported screening rates among Turkish women range from 24.5 to 48% (14–16). These findings indicate that cervical cancer screening participation remains considerably below the desired targets, underscoring the need for effective interventions to improve screening uptake.

Various intervention strategies have been developed and implemented to overcome the difficulties experienced when participating in cervical cancer screening programs (17). Nurse navigation programs have emerged as an important strategy to reduce barriers to cervical cancer screening by providing individualized guidance, education, follow-up, and support throughout the screening process (17, 18). By addressing informational, psychosocial, and practical obstacles, nurse navigation can facilitate women’s access to preventive services and strengthen their engagement in screening programs (17, 19).

Considering the preventable nature of cervical cancer and the persistent problem of low screening participation, educational interventions have been widely recommended to increase women’s awareness and promote screening behaviors (20, 21). Health education programs have been shown to improve women’s knowledge about cervical cancer and significantly increase participation in screening services (20, 22). The NNP included group-based education session, educational brochures, follow-up guidance, and supportive communication aimed at improving cervical cancer screening participation. Evidence from systematic reviews indicates that educational interventions are effective strategies for increasing cervical cancer screening uptake among women in both urban and rural settings (23).

Group education programs may be particularly effective because they allow women to receive structured information, discuss their concerns, and learn from shared experiences in a supportive environment (23, 24). These programs provide opportunities for interactive learning and peer support, which can improve women’s understanding of cervical cancer prevention and motivate them to participate in screening programs (22). Educational interventions have also been found to increase knowledge, perceived susceptibility, and motivation related to cervical cancer screening behaviors (25). Such educational approaches may help address misconceptions about cervical cancer and reduce psychological or cultural barriers associated with screening (3, 26). However, understanding women’s perceptions and experiences regarding these educational interventions is essential for improving the effectiveness and acceptability of screening promotion strategies (26).

Recent approaches to cervical cancer prevention increasingly emphasize the role of digital health technologies, mass media communication, and HPV self-sampling strategies in improving screening participation and access to preventive healthcare services (27–29). Artificial intelligence-supported health information systems may facilitate access to reliable information and increase awareness, while media campaigns and community-based communication strategies may help promote screening behaviors (27, 30). Recent evidence suggests that social media exposure may influence cervical cancer screening awareness and guideline-concordant screening behaviors (31). Furthermore, studies examining health information systems have highlighted the importance of digital platforms in facilitating access to health information, supporting communication processes, and enhancing the delivery of healthcare services. These findings underscore the growing importance of technology-supported approaches in preventive health interventions (32). In addition, HPV self-sampling has emerged as a promising approach for reducing psychosocial and structural barriers associated with cervical cancer screening (33).

The health belief model (HBM) provides a useful framework for understanding women’s cervical cancer screening behaviors by focusing on perceived susceptibility, perceived benefits, perceived barriers, and cues to action. Within this framework, psychosocial barriers, awareness, media influence, social support, and perceptions regarding HPV self-sampling may all influence women’s participation in cervical cancer screening programs. Therefore, women’s perspectives regarding these factors were also explored within the context of the NNP. Studies have shown that navigation programs incorporating health counseling, educational materials, and strategies to overcome barriers to screening significantly increase participation in cervical cancer screening compared with usual care (34, 35). However, the vast majority of existing studies have focused on quantitative outcomes, such as screening participation rates, and the mechanisms through which nurse navigation influences women’s screening behaviors have not been sufficiently elucidated. In particular, within the context of middle-income countries, women’s lived experiences, perceived benefits, and perceived barriers related to nurse navigation programs have rarely been explored using qualitative approaches (36). The aim of this study was to explore in depth the experiences of women who participated in the NNP regarding cervical cancer screening.

## 2 Materials and methods

### 2.1 Design and sample

This study was conducted as a qualitative study using thematic analysis to explore the experiences of women who participated in a NNP regarding cervical cancer screening. Thematic analysis was used to identify and interpret recurring patterns, themes, and meanings within participants’ experiences and perceptions (37, 38).

Participants consisted of women who had participated in a NNP conducted in Istanbul, Türkiye, and undergone cervical cancer screening (clinical trial number: NCT06647992). Participants were recruited from the intervention group of the previously conducted randomized controlled trial. The NNP was implemented by two members of the research team, Turkan Ozdas (TO) and Elif Donmez (ED) who were responsible for participant enrolment, intervention delivery, and follow-up throughout the intervention period. Participants’ involvement in the NNP was verified using programme records maintained throughout the intervention period, including participant tracking forms, follow-up records, and documentation of educational sessions. This study was conducted independently to explore women’s experiences and perspectives regarding cervical cancer screening behaviors. The study sample included 12 women (Table 1). Participants were selected using purposive sampling, which is widely used in qualitative studies to identify individuals who have direct experience with the phenomenon being investigated (39).

**Table 1:** General characteristics and knowledge levels of the participants before the NNP (*n* = 12).

No	Age	Marital status	Education	Knowledge of HPV testing before the NNP	Knowledge of pap smear testing before the NNP
1	54	Married	University	No	No
2	44	Married	Secondary school	Yes	Yes
3	34	Married	High school	No	No
4	35	Married	High school	Yes	Yes
5	43	Married	Secondary school	No	No
6	44	Married	High school	No	No
7	44	Married	Secondary school	No	No
8	49	Divorced	High school	Yes	Yes
9	42	Married	High school	Yes	Yes
10	39	Divorced	University	Yes	Yes
11	47	Married	High school	Yes	Yes
12	39	Divorced	High school	Yes	Yes

The inclusion criteria were: being 30–65 years of age, having participated in the nurse navigation program, and volunteering to participate in the study.

Women who had cognitive impairment that prevented participation in interviews, or refused participation were excluded from the study. In qualitative research, data collection generally continues until data saturation is reached, meaning no new information or themes emerge (40).

### 2.2 Nurse navigation program

The NNP is a comprehensive, nurse-led intervention developed and adapted to align with the cultural context and healthcare system in Türkiye. It was jointly developed by the two nurse researchers (TOz and ED) and implemented by the first author (TOz). In this study, nurse navigation refers to a nurse-led intervention that supports women throughout the cervical cancer screening process through education, counseling, reminder contacts, and assistance in overcoming barriers to screening. Consistent with the broader concept of oncology nurse navigation, which may begin before diagnosis and extend across the cancer care continuum, the NNP in this study was implemented during the prevention and early detection phases of cervical cancer care.

As part of the NNP, participants attended face-to-face educational sessions conducted in groups in a classroom setting. The educational content was developed in accordance with the DISCERN guideline and reviewed by experts. Educational sessions were supported by a structured PowerPoint presentation and brochures and included information on cervical cancer epidemiology, risk factors, symptoms, prevention strategies, HPV, screening methods, and the importance of early detection. The educational sessions lasted approximately 40 min on average, and additional time was allocated after the sessions for participants who wished to ask personal or specific questions followed by individual consultations of 10–15 min. Participants were also provided with guidance regarding access to screening services, procedural preparation, and follow-up support. In addition, nurse-led counseling and telephone follow-up were provided to all participants throughout the 6-month follow-up period. Participants were also provided with the researcher’s telephone number and were informed that they could contact the nurse navigator if they required additional information, counseling, or support regarding the screening process. All participants received the same core components of the NNP, including group-based education, counseling, reminder contacts, telephone support, and guidance regarding access to cervical cancer screening services. Follow-up contacts and counseling were provided throughout the 6-month follow-up period, including scheduled assessments at the 3rd and 6th months including group-based education, counseling, reminder contacts, telephone support, and guidance regarding access to cervical cancer screening services. Follow-up contacts and counseling were provided throughout the 6-month follow-up period, including scheduled assessments at the 3rd and 6th months personalised reminder and counselling contacts were delivered to address individual barriers and support their progress towards screening. This ongoing, individualized support distinguished the programme from a standard educational intervention.

### 2.3 Data collection

The data were collected through semi-structured interviews conducted between December 2025 and January 2026, by the researchers (TO, ED), who were trained in qualitative research methods, using a semi-structured interview guide. The semi-structured interview form was developed by the researchers based on the relevant literature, research questions and the HBM. Semi-structured interviews allow participants to freely express their experiences while enabling the researcher to guide the discussion using predetermined questions (41). The interview guide consisted of questions exploring participants’ experiences and perspectives regarding cervical cancer screening within the context of the NNP and was designed to address key HBM constructs, including perceived susceptibility, perceived severity, perceived benefits, and perceived barriers. In addition, participants were invited to share their views on emerging approaches that may support cervical cancer screening participation, including HPV self-sampling, media-based awareness strategies, and artificial intelligence–supported health information systems, such as AI-based risk assessment. NNP Prior to data collection, the interview guide was pilot tested with four women who met the study inclusion criteria. These women were not included in the final study sample. The pilot interviews were conducted to assess the clarity, comprehensibility, and appropriateness of the interview questions. No revisions were required following the pilot test, and the interview guide was used as developed for data collection. Eligible participants were contacted by telephone and invited to participate in the interviews. All invited participants agreed to participate, and no participant withdrew from the study. The interviews included questions related to knowledge and awareness about HPV and cervical cancer, the contribution of the educational sessions and brochures, psychosocial barriers affecting screening participation, individual health perceptions and behaviors, perceptions regarding HPV vaccination and HPV self-sampling, media and social influence, health system-related factors, and opinions about artificial intelligence–supported health information systems. Interviews were conducted by two researchers (TO, ED) trained in qualitative research methods and were audio-recorded with participants’ consent. Individual interviews were conducted with each participant in a quiet and private setting to ensure confidentiality. Depending on participants’ preferences and accessibility, interviews were conducted face-to-face or online through digital platforms such as Zoom or WhatsApp. Each interview lasted approximately 30–45 min. The study was designed as a single-interview qualitative study, and repeat interviews were not conducted. With participants’ permission, interviews were audio recorded and field notes were taken during the interview process to capture contextual observations. Data collection continued until data saturation was reached. Data saturation was determined through a continuous and systematic analysis of the interview data. Throughout the study, the research team regularly evaluated emerging themes and patterns to assess whether new information was still being generated. To strengthen the trustworthiness of this decision, two researchers independently examined the transcripts and reached a consensus that additional interviews were unlikely to contribute novel insights relevant to the research topic. Based on this assessment, recruitment was discontinued once saturation had been achieved, resulting in a final sample of 12 participants. All audio-recorded interviews were transcribed verbatim by the researchers (TO, ED). To maintain confidentiality, all identifying information was removed and participants were anonymized using pseudo-identifiers (e.g., P1–P12) during transcription.

### 2.4 Data analysis

The collected data were analyzed using thematic content analysis. Data analysis followed a hybrid approach, whereby themes were inductively derived from the data through thematic content analysis, while the interpretation of findings was informed by the HBM. The resulting themes were subsequently interpreted in relation to key HBM constructs, including perceived susceptibility, perceived severity, perceived benefits, and perceived barriers.

In this process, transcripts were read repeatedly to obtain an overall understanding of the data. Meaningful expressions were identified and coded, and similar codes were grouped into categories and themes that reflected participants’ experiences (38). For descriptive purposes, the number of participants refers to the number of participants whose statements were coded under a particular code, whereas the number of repetitions represents the total number of coded references identified across all interview transcripts. Because a participant could refer to the same issue multiple times during an interview, the number of repetitions may exceed the number of participants. These frequencies were reported solely to provide descriptive context and should not be interpreted as indicators of the relative importance of themes.

Two researchers (TO, ED) independently coded the data and compared the codes. Differences were discussed until consensus was reached to ensure analytical rigor. Qualitative data analysis software MAXQDA was used to facilitate data management and analysis. The trustworthiness of the data was ensured using the four criteria proposed by Lincoln and Guba: credibility, transferability, dependability, and confirmability (42). To ensure credibility, member checking was conducted before data analysis by returning the interview transcripts to participants for review and verification. Participants were invited to clarify, revise, or expand upon their responses. Minor revisions and clarifications provided by participants were incorporated into the final transcripts prior to analysis. To ensure transferability, detailed descriptions of the research setting, participant characteristics, and research procedures were provided. Dependability was ensured by maintaining an audit trail documenting all research steps and analytical decisions. To enhance confirmability, direct quotations from participants were included in the results section to represent participants’ perspectives and reduce researcher bias. The reporting of this study was guided by the COREQ (Consolidated Criteria for Reporting Qualitative Research) checklist (43).

### 2.5 Researcher characteristics and reflexivity

The research team consisted of three female academicians: an academician with a doctoral degree in oncology nursing, an associate professor in public health nursing, and a professor in public health nursing. All researchers had certificates in qualitative research methods and experience in conducting qualitative studies. The interviews were conducted by trained researchers familiar with qualitative research methods. Researchers maintained reflexive awareness throughout the study to minimize potential bias during data collection and analysis Prior to the interviews, a relationship had been established between the participants and TO through the NNP. TO, an academician with a doctoral degree in oncology nursing, was responsible for delivering the educational sessions and conducting follow-up activities throughout the intervention period. Participants were aware of TO’s role as both a researcher and the nurse navigator involved in the program. They were also informed about the roles of the other members of the research team and the purpose of the study prior to the interviews. Given TO’s involvement in the intervention, reflexive awareness was maintained throughout data collection and analysis to minimize the potential influence of prior relationships, researcher assumptions, and expectations on the interpretation of the findings (44).

### 2.6 Ethical considerations

Ethical approval for the study was obtained from the Scientific Research Ethics Committee of the University of Health Sciences (No: 597919). Written informed consent was obtained from all participants prior to the interviews. Participation was voluntary and participants were informed that they could withdraw from the study at any time without consequences. Confidentiality and anonymity were ensured throughout the research process and all procedures were conducted in accordance with the Declaration of Helsinki.

## 3 Results

Twelve women participated in the qualitative interviews. Analysis of the data led to the identification of 10 main themes, grouped under two overarching categories: Themes related to education awareness, and psychosocial barriers (Table 2), and themes related to behavior, health system, vaccination, media, and technology (Table 3).

**Table 2:** Themes related to education, knowledge, awareness, and psychosocial barriers (*n* = 12).

Themes	Codes	Number of participants	Number of repetitions
Education and awareness	Invitation attracted my interest	11	11
	Lack of knowledge before the education	11	18
	Participation to obtain information about the HPV vaccine	4	4
	Gaining new information about HPV and screening	12	26
	Understanding the necessity of screening	12	27
	Becoming more conscious	8	12
	Increased awareness after the education	12	28
	Education positively affects screening behavior	11	15
	Brochures act as reminders	12	14
Psychosocial barriers	Shame	12	46
	Embarrassment	8	15
	Fear (pain or screening results)	10	32
	Neglecting or postponing screening	11	16
	Discomfort related to gynecological examination position	6	8
	Spousal or family pressure	3	3
	Avoidance of the hospital environment	10	19

Frequencies are presented for descriptive purposes only and should not be interpreted as indicators of the relative importance of themes.

**Table 3:** Themes related to behavior, health system, vaccination, media, and technology (*n* = 12).

Themes	Codes	Number of participants	Number of repetitions
HPV self-sampling test kits	Willingness to perform the test at home	12	29
	Privacy and convenience	11	24
	Reduced embarrassment	8	11
	A solution without going to the hospital	12	27
	Belief that it will increase participation in screening	10	17
Perceptions of HPV vaccination	Cost as a barrier	10	18
	Willingness to vaccinate their children	9	10
	Belief that vaccination should be mandatory	1	1
	Positive attitude toward HPV vaccination for oneself	8	11
	Hesitation or distrust	5	7
	Hesitation about vaccinating children	5	7
Mass media influence	Awareness through television and media campaigns	11	20
	Need for visibility on social media	12	30
	Frequent exposure increases awareness	10	16
	Effect of face-to-face seminars and association meetings	12	15
Individual health perception	Routine health check-ups	5	7
	Importance of nutrition and physical activity	9	14
	Lack of time or opportunity	6	8
	Not considering screening important	6	9
Health system	Demand for free services	5	7
	Need for face-to-face seminars in rural areas	6	8
	Need for community-based education	7	11
	Suggestion of a separate screening follow-up unit in family medicine	6	9
	Perceived inadequacy of family physician follow-up	3	3
	Suggestion of home-visit-based health services	5	9
Artificial intelligence applications	Positive attitude toward AI-based health information	11	20
	Belief that AI-based systems may be ineffective	3	3
Information sharing and social influence	Sharing health information with family members	5	6
	Encouraging relatives to participate in screening	9	13
	Creating behavior change through information sharing	6	7

Frequencies are presented for descriptive purposes only and should not be interpreted as indicators of the relative importance of themes.

### 3.1 Theme 1: Education and awareness

For many participants, the invitation itself was an important factor in deciding to attend the program. Several women stated that they joined because they realised they had limited prior knowledge about HPV, cervical cancer, screening, and vaccination. Some also reported that their main motivation was to obtain more information about the HPV vaccine. As one participant explained:

“As a woman, there were some issues I did not know about, especially the vaccine and HPV test. Thanks to the education, I realised how important these issues are for me and I started paying more attention to my check-ups.” (Patient 3).

Another participant similarly noted:

“This was a very valuable activity, and it helped me become more aware. I used to feel afraid and embarrassed, and like many women, I tended to postpone screening because of hesitation. This program was very beneficial for us.” (Patient 2).

A common finding across interviews was that the education improved participants’ knowledge and awareness. All participants reported gaining new information about HPV infection and cervical cancer screening and stated that the education helped them better understand the necessity of screening. Women frequently described learning about routes of prevention and the importance of preventive health behaviors. Educational brochures distributed during the sessions were viewed positively and were often described as useful reminders that could be revisited later. One participant stated:

“We actually had no knowledge about HPV. The education was very useful for us. Receiving the booklet was also good because we had the chance to read it again and again. At least we learned what HPV is and understood the importance of screening to become healthier individuals.” (Patient 8).

Another participant similarly noted:

“This event was very beneficial for us because I had never had such detailed information about HPV before. After the education, I understood much better how important this issue is.” (Patient 6).

### 3.2 Theme 2: Psychosocial barriers

Psychosocial barriers were among the most prominent themes affecting screening behavior. All participants described feelings of shame related to cervical cancer screening, while many also reported embarrassment and fear associated with both the examination process and the possibility of receiving an abnormal result. Most women stated that they had previously neglected or postponed screening, and several participants expressed discomfort related to the gynecological examination position. Some participants also mentioned avoiding hospital environments, whereas a smaller number referred to spousal or family pressure as an additional barrier. One participant explained:

“Women may hesitate about these examinations. Honestly, I felt some shame and hesitation, so I had not undergone screening before. Since it concerns the most sensitive part of women, fear and embarrassment occur.” (Patient 3).

Another participant described how fear, embarrassment, and postponement had delayed screening participation despite previous recommendations.

“I had previously undergone an HPV test at a primary care centre, but I postponed having the test again because of work responsibilities and personal concerns. To be honest, I was also afraid, as many women feel uncomfortable about procedures involving cervical sampling. Although the nurse acted professionally, I needed encouragement and reminders before I finally decided to attend screening.” (Patient 8).

### 3.3 Theme 3: HPV self-sampling test kits

Views toward HPV self-sampling test kits were generally positive. All participants expressed willingness to perform the test at home and described home-based testing as a practical alternative to hospital-based procedures. Most women emphasized the advantages of privacy and convenience, while several participants stated that self-sampling could reduce feelings of embarrassment. Many participants also believed that self-sampling could increase participation in screening programs, particularly among women who are reluctant to attend healthcare facilities. One participant stated:

“If there were a test that could be done at home, it would be easier for many women because going to the hospital is sometimes difficult and people may feel embarrassed.” (Patient 1).

Many participants also believed that this approach could increase participation in screening programs.

Another participant emphasized that home-based testing could reduce multiple barriers associated with hospital-based screening, including embarrassment, time constraints, stress, and fear.

“Being able to obtain the test from a pharmacy and perform it at home would be a great convenience for me. Working women could easily use it, and women who are afraid or embarrassed would feel more comfortable. There would be no waiting in line, no embarrassment, and much less stress. Sometimes I cannot find time, and when I do, I worry about what the results might show. If home-based test kits were available, I would not need to make excuses or postpone screening; I could simply perform the test immediately.” (Patient 8).

### 3.4 Theme 4: Perceptions of HPV vaccination

Vaccination was generally viewed positively; however, cost emerged as a major barrier. Several women stated that they would be more likely to receive the vaccine, or vaccinate their children, if it were affordable or publicly funded. One participant noted:

“If the vaccine were free, I think more people would receive it because cost may be a barrier for some people.” (P6).

At the same time, some participants expressed uncertainty due to conflicting information from different sources:

“About a year ago, I heard many positive things about the HPV vaccine and its benefits. Later, I heard different opinions from another doctor and through social media, suggesting that the vaccine might not be useful. Now, I feel uncertain about whether vaccination is the right choice because different healthcare professionals provide different information.” (P8).

Another participant highlighted that personal concerns and a perceived lack of need for vaccination could also influence vaccination decisions.

“I have not received the vaccine. Cost is not really the issue for me. Perhaps I have not thought about it because I do not currently feel a need for it. There is also some fear involved, as I am afraid of needles. That may be another reason why I have postponed vaccination.” (P10).

### 3.5 Theme 5: Mass media influence

Mass media was widely perceived as an effective way to improve awareness. Nearly all participants reported that television and media campaigns contributed to increasing awareness about HPV and cervical cancer screening, while all participants emphasized the need for greater visibility on social media platforms. Most participants also stated that repeated exposure to health messages increased attention and awareness. In addition, all participants highlighted the positive impact of face-to-face seminars and community-based meetings in promoting awareness and encouraging participation in screening. As one participant explained:

“If these issues are explained more on social media and television, people’s awareness will increase.” (Patient 3).

Another participant emphasized that social media campaigns, celebrity involvement, and systematic follow-up strategies could further improve public awareness and participation in screening programs.

“I am not sure how feasible this would be in Türkiye, because I think social media is still insufficient in raising awareness about this issue. Since we live in a digital age, awareness campaigns similar to those used for breast cancer could be developed with the support of celebrities and healthcare professionals. I also think that women should receive follow-up reminders after being informed, so that they are encouraged to attend screening.” (Patient 12).

### 3.6 Theme 6: Individual health perception

Participants described varying levels of personal health awareness and behaviors. Some women reported engaging in routine health check-ups and being attentive to their health, while others acknowledged that they did not consistently prioritize healthy behaviors such as regular physical activity or preventive care. One participant stated:

“Normally, I take care of my health. I regularly go to the doctor when I feel something unusual. However, I have a smoking habit, and I am trying to quit it.” (Patient 3).

In contrast, another participant expressed a lower level of health awareness:

“I am not someone who pays much attention to my health. I try to walk occasionally, but not regularly. I hope I will be more careful in the future.” (Patient 5).

### 3.7 Theme 7: Health system

Participants highlighted several structural factors related to the healthcare system that influenced their screening behavior. Access to information, quality of communication with healthcare providers, and system-level organization were frequently mentioned. One participant described her previous screening experience:

“I had an HPV test years ago at a primary care centre, but I was not given detailed information. After receiving written information, education, and answers to my questions, I started to consider having the test again.” (Patient 6).

In addition, participants emphasized that the hospital environment itself could act as a barrier due to stress, waiting times, embarrassment, and time constraints:

“When I think about hospitals, I feel stressed. Making an appointment, waiting in line, and seeing other patients make me uncomfortable. I also experience embarrassment regarding gynecological examinations, and as a working woman, finding time for screening can be difficult. Sometimes I combine all of these factors and end up making excuses to postpone screening. In contrast, primary care centres feel more comfortable and welcoming. The friendly approach of healthcare professionals reduces my fear and stress, making me more willing to consider screening.” (Patient 8).

### 3.8 Theme 8: Artificial intelligence applications

Nearly all participants expressed positive attitudes toward the use of artificial intelligence in health education and information delivery. AI-based systems were generally perceived as practical and potentially useful tools for improving access to reliable health information. Participants emphasized that artificial intelligence could facilitate access to information, increase awareness, and potentially encourage screening participation However, a few participants believed that AI-based systems might be less effective than direct communication with healthcare professionals. Participants stated:

“Artificial intelligence is already part of our lives and helps us in many areas. It can also be used for health information.” (Patient 11).

“I think it would have a great impact because we all use artificial intelligence now. Even for minor illnesses, we ask AI questions and receive detailed explanations. We could also learn more about cervical cancer through such systems.” (Patient 3).

“If an AI-based system indicated that I might be at risk, I would definitely go for screening. At the moment, I do not actively seek information or think about it, but such a warning would motivate me to take action.” (Patient 2).

### 3.9 Theme 9: Information sharing and social influence

Several participants reported sharing health-related information with family members and people in their close social circles following the education. Many women stated that they encouraged their relatives and friends to participate in cervical cancer screening. Some participants also expressed that sharing information contributed to increasing awareness and promoting behavioral change among others. One participant said:

“After this education, I started telling the women around me that they should have screening. I told my mother and my sister.” (Patient 2).

Another participant emphasized that the education stimulated discussions among women and encouraged mutual support for screening.

“It definitely changed my awareness. After this seminar, women talked about these issues much more. We naturally encouraged one another to undergo screening and participate in preventive practices.” (Patient 7).

Another participant also emphasized encouraging others:

“I recommended it to others. It is quite effective. For example, when we sit together, I tell them ‘I had it done, you should also have it done,’ and we definitely encourage each other.” (Patient 9).

## 4 Discussion

This study explored women’s perspectives on the impact of the NNP on their cervical cancer screening behaviors. The findings indicate that, although the intervention substantially improved knowledge and awareness, screening behavior continues to be shaped by a complex interplay of psychosocial, individual, and system-level factors.

One of the most notable findings was the significant improvement in participants’ knowledge and awareness following the NNP.

These findings are consistent with the perceived benefits and perceived susceptibility constructs of the HBM. The NNP appeared to increase women’s awareness of cervical cancer screening and contribute to the adoption of preventive health behaviors. Beyond improving knowledge, the program may also have influenced how women perceived cervical cancer screening. Participants frequently reported becoming more aware of the importance of screening, feeling more confident about engaging with preventive health services, and expressing greater willingness to undergo screening after participating in the program. These findings suggest that navigation-based support may increase the perceived benefits of screening while reducing concerns and barriers that discourage participation. Such changes in perceptions and intentions may facilitate engagement in cervical cancer screening and are consistent with previous evidence demonstrating that patient navigation interventions improve screening uptake through education, individualized support, and assistance in overcoming barriers to care (17–19, 34).

Many women reported having limited prior knowledge about HPV, vaccination, and screening, and indicated that the education helped them better understand the importance of preventive health behaviors. This finding is consistent with previous research demonstrating that structured educational interventions significantly enhance awareness and increase participation in screening. This result is consistent with the findings of Fricovsky et al. (45); their study demonstrated that educational seminars increased women’s knowledge of HPV, Pap smear, and cervical cancer, and strengthened their intention to undergo screening. Similarly, other studies have reported that educational interventions can lead to substantial improvements in awareness, with increases exceeding 200% among young women following education (46). In a systematic review conducted by Zhang et al. (23), it was reported that educational programs similar to this study, conducted in the form of community-based group sessions and delivered by healthcare professionals using culturally sensitive materials—increased women’s knowledge levels and cervical cancer screening behaviors. A meta-analysis found that, based on a review of 9 studies, educational approaches increased screening rates and acceptance by approximately threefold (21). The educational sessions, brochures, and media exposure acted as cues to action by prompting women to seek information and participate in cervical cancer screening. This finding is consistent with the HBM, which identifies external prompts and reminders as important triggers of preventive health behaviors (14). The findings of both the present study and previous research consistently demonstrate that navigation-based educational interventions are effective in improving women’s knowledge and awareness regarding HPV infection, cervical cancer prevention, vaccination, and participation in screening programs. These results highlight the important role of culturally tailored education and patient navigation strategies in enhancing preventive health behaviors and facilitating engagement with cervical cancer screening services, particularly among populations with limited baseline awareness and barriers to healthcare access.

In this study, many participants reported that psychosocial barriers such as shame, embarrassment, and fear continued to strongly influence their screening behaviors despite increased awareness. These findings align with a substantial body of literature emphasizing that emotional and sociocultural factors continue to hinder participation in cervical cancer screening programs. The intimate nature of gynecological examinations, when combined with culturally embedded norms of modesty and privacy, may lead women to avoid or delay screening. Beyond functioning as independent barriers, fear and shame appeared to interact and reinforce one another. Many women reported avoiding screening not only because of concerns about a possible cancer diagnosis but also because of embarrassment related to gynecological examinations. These psychosocial barriers were further amplified by negative perceptions of healthcare settings and difficulties accessing services. Together, these findings suggest that cervical cancer screening behavior is influenced by the combined effects of emotional and structural barriers rather than by a single factor alone (10, 11, 15, 47, 48). Within the HBM framework, these findings reflect the perceived barriers construct, highlighting that shame, fear, and avoidance remain significant obstacles to cervical cancer screening participation.

Consistent with previous studies, women frequently report fear related not only to the procedure itself including concerns about pain, the use of the speculum, and the clinical environment but also to the possibility of receiving a cancer diagnosis or an unfavorable result (10, 11). These fears reflect both procedural anxiety and deeper existential concerns, which may significantly reduce screening uptake. Furthermore, sociocultural context plays a critical role in shaping these perceptions. Studies conducted among migrant women in Europe indicate that cultural and religious values, particularly those related to modesty, honor, and privacy, may contribute to perceiving pelvic examinations as intrusive, degrading, or even threatening. In some groups, examination by a male physician is considered unacceptable, further limiting access to screening services (49). Similarly, deeply rooted norms surrounding genital privacy and bodily reserve have been shown to render pelvic examinations culturally inappropriate or uncomfortable, thereby fostering avoidance or strong reluctance toward screening participation (47, 48, 50). Taken together, these findings suggest that cervical cancer prevention strategies should extend beyond knowledge-based interventions and incorporate culturally sensitive, gender-responsive, and psychologically supportive approaches to effectively address these persistent barriers.

In this study, system-level barriers were also found to play an important role in shaping screening behavior in addition to individual and psychosocial factors. Participants identified factors such as insufficient information provided during healthcare encounters, inadequate follow-up mechanisms, time constraints, and the stressful nature of hospital environments as key barriers. From a HBM perspective, structural and systemic inadequacies represent perceived barriers that continue to limit women’s access to and participation in screening services. These findings are consistent with studies in the literature highlighting the impact of structural problems within healthcare systems on participation in screening services. In a study conducted by Owokuhaisa et al. (51), long waiting times, overcrowding, lack of privacy, delays in laboratory results, and insufficient staffing were reported to negatively affect participation in healthcare services and continuity of care. Similarly, in a scoping review examining the participation of underserved groups in cervical cancer screening in Europe, Greenley et al. (9) identified difficulties in registering with primary care providers, challenges in obtaining required documents, inaccurate patient contact information, and inadequate invitation/reminder systems as significant barriers. Petersen et al. (10) emphasized that insufficient, incorrect, or absent information regarding screening and follow-up processes constitutes a recurring barrier at individual, interpersonal, and system levels. In addition, Kassa et al. (52) reported that distance, transportation costs, inconvenient service hours, and inaccessible healthcare facilities—particularly for women with disabilities—negatively influence participation in screening programs. These findings suggest that improving communication within healthcare services, strengthening follow-up systems, increasing accessibility, and developing more patient-centred and supportive screening services may contribute to improving women’s participation in cervical cancer screening programs.

The findings of this study showed that participants generally had positive views toward HPV self-sampling test kits. Participants described home-based testing as practical, convenient, and time-saving. These findings are consistent with the systematic review by Nishimura et al. (53), which reported that women preferred HPV self-sampling because it provides privacy, convenience, and reduces the need for hospital visits. The review also emphasized that home-based sampling may increase participation in cervical cancer screening, especially among women who do not regularly attend screening programs. Similar findings were reported in the scoping review by Colonetti et al. (54), which highlighted self-sampling as an acceptable and feasible approach that may improve participation in cervical cancer screening programs. In addition, the meta-analysis by Yeh et al. (55) found that most women preferred self-sampling over clinician-collected samples and that self-sampling nearly doubled cervical cancer screening uptake compared with standard screening invitations. Participants’ willingness to undergo screening and their positive perceptions of HPV self-sampling may also reflect increased self-efficacy regarding cervical cancer prevention. This finding is consistent with the HBM, which suggests that individuals who feel confident in their ability to perform a health-related behavior are more likely to engage in preventive actions (14).

A separate theme that emerged from the findings was participants’ perceptions of artificial intelligence–supported health information systems. Most participants expressed positive views regarding the potential use of AI in health education and information delivery. Participants considered AI-based applications practical and useful for increasing access to reliable health information and supporting screening-related decision-making. These findings are consistent with the review by Galani et al. (28), which highlighted the potential role of artificial intelligence–supported technologies in cervical cancer screening strategies, risk assessment, and personalized preventive healthcare in the digital era. It should be noted, however, that participants discussed hypothetical AI-supported risk assessment and screening reminder systems rather than reporting direct experience with specific AI-based health applications. Therefore, these findings reflect participants’ perceptions and expectations regarding the potential role of AI in cervical cancer screening rather than actual experiences with AI-supported health technologies.

In this study, individual health perception emerged as an important factor influencing cervical cancer screening behavior. While some participants described engaging in routine health check-ups and paying attention to nutrition and physical activity, others reported limited attention to preventive health behaviors and stated that they did not always consider screening to be important. In this study, it was found that individual perceptions of health are one of the key factors influencing cervical cancer screening behavior. While some participants reported attending regular health checkups and paying attention to their diet and physical activity, others stated that they did not place sufficient importance on preventive health behaviors and did not always consider screening to be important. Similarly, a study by Aslan et al. (56) reported that light physical activity, healthier dietary habits, and living in the Mediterranean region positively influenced participation in cervical cancer screening. In addition, lack of time and limited opportunities were frequently cited as barriers to participation in screening programs. These findings suggest that women’s individual perceptions of health and lifestyle behaviors may influence their participation in preventive health services. Studies conducted in low- and middle-income countries also report, similarly to the findings of this study, that women experience time constraints due to family responsibilities and household chores and are unable to allocate sufficient time to their own health needs. These findings suggest that women’s individual health perceptions and lifestyle behaviors may influence their participation in preventive health services (57, 58).

The findings of this study also highlighted the importance of information sharing and social influence in the formation of browsing behavior. Several participants reported sharing health-related information with family members and encouraging relatives or friends to participate in cervical cancer screening. Some women also stated that information sharing contributed to increasing awareness and promoting behavioral change within their social environments. These findings suggest that social interaction and peer influence may play an important role in the dissemination of preventive health behaviors. Qualitative studies conducted in Sub-Saharan Africa indicate that recommendations or encouragement from friends and relatives motivate women to undergo screening; the likelihood of participating in screening increases when “model” protective behaviors are observed among peers or relatives. The same study emphasizes that sharing screening experiences among relatives and peers increases knowledge, reduces fear and stigma, and boosts willingness to undergo screening. A systematic qualitative review also reports that strong social networks facilitate access to referrals and screenings within the healthcare system, particularly for newcomers or disadvantaged women (59). Wagner et al. (60) note that the intervention not only led the “trained” women to begin advocating for screening among others but also prompted their peers to do the same, highlighting the potential for this to spread throughout the entire community over time.

The themes related to HPV vaccination, information sharing, media exposure, and artificial intelligence applications suggest that women’s decisions regarding cervical cancer prevention are shaped not only by individual knowledge but also by broader social and informational influences. Participants frequently emphasized the role of family members, social networks, media campaigns, and digital information sources in shaping their awareness and attitudes. These findings indicate that cervical cancer prevention efforts may be strengthened through approaches that combine individual education with community-based communication strategies and technology-supported health information systems (28, 29, 60). Overall, the findings suggest that the NNP may support cervical cancer screening participation by addressing multiple factors that influence screening behavior, including knowledge, perceived barriers, social influences, and access-related challenges. A notable contribution of the present study is that it provides an integrated understanding of women’s perceptions of multiple strategies related to cervical cancer screening participation within a single screening context. While previous studies have often examined screening barriers, nurse-led education, HPV self-sampling, media-based interventions, social influences, or artificial intelligence-supported health information separately (19, 28, 55, 60), the present study explored how women perceived these approaches together following participation in a NNP. Participants described nurse-led education, reminder-based follow-up, HPV self-sampling, media communication, and AI-supported health information as complementary strategies that may help address different psychosocial, informational, and health system-related barriers to cervical cancer screening. By examining these interconnected strategies simultaneously, the study offers a broader perspective on how combined educational, behavioral, technological, and social approaches may support participation in cervical cancer screening. This integrated perspective contributes to the growing literature on multidimensional and technology-supported approaches to cervical cancer screening participation.

### 4.1 Strengths and limitations

This study has several strengths. First, it provides an in-depth examination of women’s experiences and perspectives regarding cervical cancer screening within the context of a NNP. Second, the inclusion of psychosocial, social, technological, and health system-related factors has enabled a comprehensive assessment of screening behaviors. Additionally, the use of thematic analysis, direct participant quotes, adherence to the HBM, and the COREQ checklist has strengthened the study’s methodological rigor.

However, certain limitations should be considered. Participants were selected exclusively from women who had participated in a NNP and agreed to in-depth interviews; this may have introduced selection bias. Furthermore, since most findings are consistent with the existing literature, the possibility of confirmation bias should also be considered. Furthermore, the findings reflect the experiences of women within a specific cultural and geographical context and may therefore not be generalizable to all populations.

## 5 Conclusion

This study demonstrates that women’s cervical cancer screening behaviors are influenced by various factors, including psychosocial, social, technological, individual, and health system-related factors. The findings suggest that participants perceived the NNP as contributing to increased knowledge and awareness regarding HPV, cervical cancer, and screening. NNP; However, participants also reported that barriers such as fear, shame, embarrassment, lack of time, and stressful healthcare environments continued to influence their screening-related decisions and behaviors. Positive perceptions of self-sampling methods for HPV, media-based awareness strategies, and AI-supported health information systems suggest that innovative and accessible approaches can support screening participation.

The findings also emphasize that community-based education, supportive communication, and accessible healthcare services play a significant role in improving cervical cancer screening behaviors. Participants perceived that the NNP helped them address barriers related to cervical cancer screening through education, guidance, counselling, and follow-up support.

NNP It is recommended that future interventions adopt multidimensional approaches addressing both individual and structural barriers and incorporate culturally sensitive, community-based, and technology-supported strategies to increase participation in cervical cancer screening programs.

## Data Availability

The original contributions presented in the study are included in the article/supplementary material, further inquiries can be directed to the corresponding author.

## References

[1] World Health Organization. (2025). Cervical cancer. Available online at: https://www.who.int/news-room/fact-sheets/detail/cervical-cancer (Accessed May 16, 2026).

[2] Bray F Laversanne M Sung H Ferlay J Siegel RL Soerjomataram I . Global cancer statistics 2022: GLOBOCAN estimates of incidence and mortality worldwide for 36 cancers in 185 countries. CA Cancer J Clin. (2024) 74:229–63. doi: 10.3322/caac.21834, 38572751

[3] Arbyn M Weiderpass E Bruni L de Sanjosé S Saraiya M Ferlay J . Estimates of incidence and mortality of cervical cancer in 2018: a worldwide analysis. Lancet Global Health. (2020) 8:e191–203. doi: 10.1016/S2214-109X(19)30482-6, 31812369 PMC7025157

[4] Horn J Denecke A Luyten A Rothe B Reinecke-Lüthge A Mikolajczyk R . Reduction of cervical cancer incidence within a primary HPV screening pilot project (WOLPHSCREEN) in Wolfsburg. Germany Br J Cancer. (2019) 120:1015–22. doi: 10.1038/s41416-019-0453-2, 30988395 PMC6734660

[5] Jansen EEL Zielonke N Gini A Anttila A Segnan N Vokó Z . Effect of organised cervical cancer screening on cervical cancer mortality in Europe: a systematic review. Eur J Cancer. (2020) 127:207–23. doi: 10.1016/j.ejca.2019.12.013, 31980322

[6] Bruni L Serrano B Roura E Alemany L Cowan M Herrero R . Cervical cancer screening programmes and age-specific coverage estimates for 202 countries and territories worldwide: a review and synthetic analysis. Lancet Global Health. (2022) 10:e1115–27. doi: 10.1016/S2214-109X(22)00241-8, 35839811 PMC9296658

[7] National Cancer Institute. ACS’S updated cervical cancer screening guidelines explained. (2020). Available online at: https://www.cancer.gov/news-events/cancer-currents-blog/2020/cervical-cancer-screening-hpv-test-guideline (Accessed January 6, 2025).

[8] Sung H Ferlay J Siegel RL Laversanne M Soerjomataram I Jemal A . Global cancer statistics 2020: GLOBOCAN estimates of incidence and mortality worldwide for 36 cancers in 185 countries. CA Cancer J Clin. (2021) 71:209–49. doi: 10.3322/caac.21660, 33538338

[9] Greenley R Bell S Rigby S Legood R Kirkby V McKee M . Factors influencing the participation of groups identified as underserved in cervical cancer screening in Europe: a scoping review of the literature. Front Public Health. (2023) 11:1144674. doi: 10.3389/fpubh.2023.1144674, 37304105 PMC10247980

[10] Petersen Z Jaca A Ginindza TG Maseko G Takatshana S Ndlovu P . Barriers to uptake of cervical cancer screening services in low- and middle-income countries: a systematic review. BMC Womens Health. (2022) 22:486. doi: 10.1186/s12905-022-02043-y, 36461001 PMC9716693

[11] Srinath A Van Merode F Rao SV Pavlova M. Barriers to cervical cancer and breast cancer screening uptake in low- and middle-income countries: a systematic review. Health Policy Plan. (2023) 38:509–27. doi: 10.1093/heapol/czac104, 36525529 PMC10089064

[12] Gultekin M Zayifoglu Karaca M Kucukyildiz I Dundar S Boztas G Semra Turan H . Initial results of population based cervical cancer screening program using HPV testing in one million Turkish women. Int J Cancer. (2018) 142:1952–8. doi: 10.1002/ijc.31212, 29235108 PMC5888190

[13] Özdemir R Türkmen Çevik F Kes D Karacali M Özgüner S. Level and factors associated with participation in population-based cancer screening in Safranbolu district of Karabuk. Turkey Iran J Public Health. (2020) 49:663–72. doi: 10.18502/ijph.v49i4.3172, 32548046 PMC7283187

[14] Kırag N. Knowledge, health beliefs, and preventive behavior regarding cervical cancer among Turkish women: a cross sectional study. J Public Health (Berl). (2021) 29:239–44. doi: 10.1007/s10389-020-01382-0

[15] Çankara S Yüksel G. Barriers to women’s pap smear testing and related risk factors in Turkey. Clin Exp Health Sci. (2020) 10:203–9. doi: 10.33808/marusbed.645170

[16] Koç O Baltacı N Yüksekol DO. The relationship of women's cervical cancer screening beliefs with their beliefs on HPV vaccine. TJFMPC. (2023) 17:31–41. doi: 10.21763/tjfmpc.1119611

[17] Nelson HD Cantor AG Pappas M Blackie K Yu Y Fu R. Patient navigation services for breast and cervical cancer screening and follow-up: a meta-analysis. JAMA Intern Med. (2025) 185:976–86. doi: 10.1001/jamainternmed.2025.1590 40489104 PMC12150228

[18] Nelson HD Cantor A Wagner J Jungbauer R Fu R Kondo K . Effectiveness of patient navigation to increase cancer screening in populations adversely affected by health disparities: a meta-analysis. J Gen Intern Med. (2020) 35:3026–35. doi: 10.1007/s11606-020-06020-9 32700218 PMC7573022

[19] Mosquera I Todd A Balaj M Zhang L Benitez Majano S Mensah K . Components and effectiveness of patient navigation programmes to increase participation to breast, cervical and colorectal cancer screening: a systematic review. Cancer Med. (2023) 12:14584–611. doi: 10.1002/cam4.6050 37245225 PMC10358261

[20] Agide FD Garmaroudi G Sadeghi R Shakibazadeh E Yaseri M Koricha ZB . A systematic review of the effectiveness of health education interventions to increase cervical cancer screening uptake. Eur J Pub Health. (2018) 28:1156–62. doi: 10.1093/eurpub/cky197 30346504

[21] Akinola A Constance MS. Impact of educational intervention on cervical cancer screening uptake among reproductive age women. Int J Community Med Public Health. (2021) 8:2053–9. doi: 10.18203/2394-6040.ijcmph20211280

[22] Saei Ghare Naz M Kariman N Ebadi A Ozgoli G Ghasemi V Rashidi F. Educational interventions for cervical cancer screening behavior of women: a systematic review. Asian Pac J Cancer Prev. (2018) 19:875–84. doi: 10.22034/APJCP.2018.19.4.875 29693331 PMC6031778

[23] Zhang M Sit JWH Chan DNS Akingbade O Chan CWH. Educational interventions to promote cervical cancer screening among rural populations: a systematic review. Int J Environ Res Public Health. (2022) 19:6874. doi: 10.3390/ijerph19116874 35682457 PMC9180749

[24] Mariño JM Nunes LM Ali YC Tonhi LD Salvetti MD. Educational interventions for cervical cancer prevention: a scoping review. Rev Bras Enferm. (2023) 76:e20230018. doi: 10.1590/0034-7167-2023-0018 38018622 PMC10680385

[25] Al-Oseely S Abdul Manaf R Ismail S. Factors affecting cervical cancer screening among Yemeni immigrant women in Klang Valley, Malaysia: a cross sectional study. PLoS One. (2023) 18:e0290152. doi: 10.1371/journal.pone.0290152 38100481 PMC10723656

[26] Marlow LAV Chorley AJ Haddrell J Ferrer R Waller J. Understanding the heterogeneity of cervical cancer screening non-participants: data from a national sample of British women. Eur J Cancer. (2017) 80:30–8. doi: 10.1016/j.ejca.2017.04.017 28535495 PMC5489076

[27] Costa T Bateson D Woo YL. Enhancing equity in cervical screening – initiatives to increase screening participation. Curr Opin Obstet Gynecol. (2024) 36:345–52. doi: 10.1097/GCO.0000000000000979 39109609

[28] Galani A Zikopoulos A Moustakli E Potiris A Paraskevaidi M Arkoulis I . Cervical cancer screening in the HPV-vaccinated and digital era: reassessing strategies in light of artificial intelligence and evolving risk. Cancers (Basel). (2025) 17:3179. doi: 10.3390/cancers17193179 41097708 PMC12524006

[29] Gomes M Provaggi E Pembe AB Olaitan A Gentry-Maharaj A. Advancing cervical cancer prevention equity: innovations in self-sampling and digital health technologies across healthcare settings. Diagnostics (Basel). (2025) 15:1176. doi: 10.3390/diagnostics15091176 40361993 PMC12071443

[30] Smith JS Vaz OM Gaber CE Marais ACD Chirumamilla B Hendrickson L . Recruitment strategies and HPV self-collection return rates for under-screened women for cervical cancer prevention. PLoS One. (2023) 18:e0280638. doi: 10.1371/journal.pone.0280638 36952486 PMC10035812

[31] Zheng F Wang K. The impact of social media on guideline-concordant cervical cancer-screening: insights from a national survey. Public Health. (2023) 223:50–6. doi: 10.1016/j.puhe.2023.07.025 37598576

[32] Zheng F Wang K Wang Q Yang Y Luo Y Zhang Y . Factors influencing clinicians' use of hospital information systems for infection prevention and control: cross-sectional study based on the extended DeLone and McLean model. J Med Internet Res. (2023):25. doi: 10.2196/44900 PMC10337337 37347523

[33] Glass-Riveros E Baumann K Craemer K Geller S Nava Frenier M McDonald J . The acceptability and feasibility of self-collected HPV testing for cervical cancer screening among black and Latinx women in Chicago: perspectives from the community. Womens Health Rep (New Rochelle). (2024) 5:whr.2024.0102. doi: 10.1089/whr.2024.0102 PMC11512086 39463469

[34] Mann K Sun K Jang J Khilnani A Barrett E Harrod T . Patient navigation as an effective way to increase breast, cervical, and colorectal cancer screening among immigrants in the US: a systematic review. J Clin Oncol. (2024) 42:e22501. doi: 10.1200/JCO.2024.42.16_suppl.e22501 PMC12344048 40329031

[35] Wang X Fang C Tan Y Liu A Ma GX. Evidence-based intervention to reduce access barriers to cervical cancer screening among underserved Chinese American women. J Womens Health (Larchmt). (2010) 19:463–9. doi: 10.1089/jwh.2009.1422 20156089 PMC2867551

[36] Akoto EJ Allsop MJ. Factors influencing the experience of breast and cervical cancer screening among women in low- and middle-income countries: a systematic review. JCO Global Oncol. (2023) 9:e2200359. doi: 10.1200/GO.22.00359 PMC10281451 37141559

[37] Ahmed SK Mohammed RA Nashwan AJ Ibrahim RH Abdalla AQ Ameen BM . Using thematic analysis in qualitative research. J Med Surg Public Health. (2025) 6:100198. doi: 10.1016/j.glmedi.2025.100198

[38] Braun V Clarke V. Using thematic analysis in psychology. Qual Res Psychol. (2006) 3:77–101. doi: 10.1191/1478088706qp063oa

[39] Donmez E. "Nitel araştırma". In: Mete B Demirhindi H, editors. Sağlık Bilimlerinde Araştırma Yöntemleri: Epidemiyolojide Araştırma Tasarımları ve Özel Konular. Ankara: Akademisyen Kitabevi (2025). p. 205–35.

[40] Guest G Bunce A Johnson L. How many interviews are enough? An experiment with data saturation and variability. Field Methods. (2006) 18:59–82. doi: 10.1177/1525822X05279903

[41] Kallio H Pietilä AM Johnson M Kangasniemi M. Systematic methodological review: developing a framework for a qualitative semi-structured interview guide. J Adv Nurs. (2016) 72:2954–65. doi: 10.1111/jan.13031 27221824

[42] Lincoln YS Guba EG. Naturalistic Inquiry. Newbury Park, CA: SAGE Publications (1985).

[43] Tong A Sainsbury P Craig J. Consolidated criteria for reporting qualitative research (COREQ): a 32-item checklist for interviews and focus groups. Int J Qual Health Care. (2007) 19:349–57. doi: 10.1093/intqhc/mzm042 17872937

[44] Creswell JW. Qualitative Inquiry and Research Design: Choosing among Five Approaches. 5th ed. Thousand Oaks, CA: SAGE Publications (2024).

[45] Fricovsky E Arain MI Tran B Nguyen PT Phan T Chang N. Assessing the impact of a health education outreach project on cervical cancer awareness among Vietnamese-American women in San Diego. AIMS Public Health. (2022) 9:552–8. doi: 10.3934/publichealth.2022038 36330281 PMC9581738

[46] Sadoh AE Okonkwo C Nwaneri DU Ogboghodo BC Eregie CO Oviawe O . Effect of peer education on knowledge of human papilloma virus and cervical cancer among female adolescent students in Benin City, Nigeria. Ann Glob Health. (2018) 84:121–28. doi: 10.29024/aogh.24 30873785 PMC6748253

[47] Fulane G Major M Lorenzoni C. Living in the minds of others: how Pudor and social judgements affect women’s cervical cancer screening uptake in Mozambique. Health Plann Manag. (2025) 40:381–90. doi: 10.1002/hpm.3881 39658457

[48] Shariati-Sarcheshme M Mahdizdeh M Tehrani H Jamali J Vahedian-Shahroodi M. Women’s perception of barriers and facilitators of cervical cancer pap smear screening: a qualitative study. BMJ Open. (2024) 14:e072954. doi: 10.1136/bmjopen-2023-072954 PMC10806721 38191254

[49] Marques P Nunes M Antunes MDL Heleno B Dias S. Factors associated with cervical cancer screening participation among migrant women in Europe: a scoping review. Int J Equity Health. (2020) 19:160. doi: 10.1186/s12939-020-01275-4 32917224 PMC7488650

[50] Isa Modibbo F Dareng E Bamisaye P Jedy-Agba E Adewole A Oyeneyin L . Qualitative study of barriers to cervical cancer screening among Nigerian women. BMJ Open. (2016) 6:e008533. doi: 10.1136/bmjopen-2015-008533 PMC4716205 26754174

[51] Owokuhaisa J Turyakira E Ssedyabane F Tusubira D Kajabwangu R Musinguzi P . Barriers and facilitators of retention in care after cervical cancer screening: patients’ and healthcare providers’ perspectives. BMC Womens Health. (2024) 24:516. doi: 10.1186/s12905-024-03343-1 39272088 PMC11401415

[52] Kassa RN Shifti DM Alemu K Omigbodun AO. Integration of cervical cancer screening into healthcare facilities in low- and middle-income countries: a scoping review. PLoS Global Public Health. (2024) 4:e0003183. doi: 10.1371/journal.pgph.0003183 38743652 PMC11093339

[53] Nishimura H Yeh PT Oguntade H Kennedy CE Narasimhan M. HPV self-sampling for cervical cancer screening: a systematic review of values and preferences. BMJ Global Health. (2021) 6:e003743. doi: 10.1136/bmjgh-2020-003743 PMC8137189 34011537

[54] Colonetti T Uggioni MLR dos SALM Uggioni NM Elibio LU Balbinot EL . Self-sampling for HPV testing in cervical cancer screening: a scoping review. Eur J Obstet Gynecol Reprod Biol. (2024) 296:20–51. doi: 10.1016/j.ejogrb.2024.02.032 38394715

[55] Yeh PT Kennedy CE de Vuyst H Narasimhan M. Self-sampling for human papillomavirus (HPV) testing: a systematic review and meta-analysis. BMJ Global Health. (2019) 4:e001351. doi: 10.1136/bmjgh-2018-001351 PMC6529022 31179035

[56] Aslan B Önal Ö. Factors influencing the decision to undergo cervical cancer screening tests (pap smear and HPV-DNA testing) in women aged 30–65: the role of HPV knowledge, cancer screening perception, health anxiety, and behaviors for protection against sexually transmitted diseases. J Health Psychol. (2026) 31:1898–914. doi: 10.1177/13591053251376855 41044882

[57] Devarapalli P Labani S Nagarjuna N Panchal P Asthana S. Barriers affecting uptake of cervical cancer screening in low and middle income countries: a systematic review. Indian J Cancer. (2018) 55:318–26. doi: 10.4103/ijc.IJC_253_18 30829264

[58] Mantula F Toefy Y Sewram V. Barriers to cervical cancer screening in Africa: a systematic review. BMC Public Health. (2024) 24:525. doi: 10.1186/s12889-024-17842-1 38378542 PMC10877795

[59] Atnafu DD Khatri R Assefa Y. Drivers of cervical cancer prevention and management in sub-Saharan Africa: a qualitative synthesis of mixed studies. Health Res Policy Syst. (2024) 22:21. doi: 10.1186/s12961-023-01094-3 38331830 PMC10851545

[60] Wagner GJ Matovu JKB Juncker M Namisango E Bouskill K Nakami S . Effects of a peer advocacy intervention on cervical cancer screening among social network members: results of a randomized controlled trial in Uganda. J Behav Med. (2023) 46:930–9. doi: 10.1007/s10865-023-00418-6 37702912 PMC10577098

